# Re-irradiation for oligo-recurrence from esophageal cancer with radiotherapy history: a multi-institutional study

**DOI:** 10.1186/s13014-017-0882-0

**Published:** 2017-09-05

**Authors:** Keiichi Jingu, Yuzuru Niibe, Hideomi Yamashita, Kuniaki Katsui, Toshihiko Matsumoto, Tomohiro Nishina, Atsuro Terahara

**Affiliations:** 1 Department of Radiation Oncology, Tohoku University Graduate School of Medicine, 1-1 Seiryo-chou, Aoba-ku, Sendai, 980-8574 Japan; 2 Department of Radiology, Toho University Omori Medical Center, Tokyo, Japan; 3 Department of Radiology, the University of Tokyo, Tokyo, Japan; 4 Department of Proton Beam Therapy, Okayama University, Okayama, Japan; 5 Department of Internal Medicine, Himeji Red Cross Hospital, Himeji, Japan; 6 Department of Gastrointestinal Medicine, Shikoku Cancer Center, Matsuyama, Japan

**Keywords:** Oligo-recurrence, Chemoradiotherapy, Re-irradiation, Esophageal cancer

## Abstract

**Background:**

Neoadjuvant chemoradiotherapy following surgery has recently become a standard therapy. The purpose of the present study was to determine the effectiveness and toxicity of re-irradiation for oligo-recurrence in lymph nodes from esophageal cancer treated by definitive radiotherapy or by surgery with additional radiotherapy.

**Methods:**

We reviewed retrospectively 248 patients treated with (chemo)radiotherapy for oligo-recurrence in lymph nodes from esophageal cancer in five Japanese high-volume centers between 2000 and 2015. Thirty-three patients in whom re-irradiation was performed were enrolled in this study, and the results for patients in whom re-irradiation was performed were compared with the results for other patients.

**Results:**

Median maximum lymph node diameter was 22 mm. Median total radiation dose was 60 Gy. The median calculated biological effective dose using the LQ model with α/β = 10 Gy (BED10) in patients in whom re-irradiation was performed was significantly lower than the median BED10 in others. There was no different factor except for BED10, histology and irradiation field between patients with a past irradiation history and patients without a past irradiation history. The median observation period in surviving patients in whom re-irradiation was performed was 21.7 months. The 3-year overall survival rate in the 33 patients with a past irradiation history was 17.9%, with a median survival period of 16.0 months. Overall survival rate and local control rate in patients with a past irradiation history were significantly worse than those in patients without a past irradiation history (log-rank test, *p* = 0.016 and *p* = 0.0007, respectively). One patient in whom re-irradiation was performed died from treatment-related gastric hemorrhage.

**Conclusions:**

Results in the present study suggested that re-irradiation for oligo-recurrence in lymph nodes from esophageal cancer treated by definitive radiotherapy or by surgery with additional radiotherapy might be acceptable but unsatisfactory.

## Background

Cancer status with ≤ 5 metastatic or recurrent lesions and with controlled primary lesions can be considered as “oligo-recurrence”. The concept of oligo-recurrence was proposed by Niibe et al. [1, 2]. Our study group has reported that oligo-recurrence in lymph nodes from esophageal cancer can be cured by radiotherapy, especially chemoradiotherapy [2]. Surgery alone has so far been a standard treatment method for primary esophageal cancer. Therefore, in patients with oligo-recurrence in regional lymph nodes, definitive radiotherapy could be performed relative safely with good results [2, 3]. However, neoadjuvant chemoradiotherapy following surgery has recently become a standard therapy [4]. There have been no report showing the efficacy and safety of re-irradiation for lesions with a radiotherapy history in patients with esophageal cancer.

The purpose of the present study was to determine the effectiveness and toxicity of re-irradiation for oligo-recurrence in lymph nodes from esophageal cancer treated by definitive radiotherapy or by surgery with additional radiotherapy.

## Methods

We reviewed retrospectively 248 patients who received (chemo)radiotherapy for oligo-recurrence in lymph nodes from esophageal cancer in 5 Japanese high-volume centers between 2000 and 2015.

The eligibility criteria for this retrospective analysis were as follows: a) the primary lesion of esophageal cancer was controlled; b) having 1–5 lymph nodes recurrences; c) without recurrence other than lymph node; and d) salvage radiotherapy or chemoradiotherapy for lymph node recurrence was given.

Of those 248 patients, 33 patients in whom re-irradiation was performed were enrolled in this study, and the results for patients in whom re-irradiation was performed were compared with the results for other patients.

The disease-free interval (DFI) was defined as the interval between initial therapy for the primary lesion and the date of identification of recurrence.

### Toxicity

Toxicity was graded according to the Common Terminology Criteria for Adverse Events (CTCAE v4.0).

### Statistical analysis

Survival estimates were calculated using the Kaplan-Meier method from the first date of radiotherapy for oligo-recurrence, and differences were evaluated by the log-rank test.

A *P* value of less than 0.05 was considered significant. All analyses were performed using IBM Statistical Package for Social Sciences (SPSS), version 22.0.

## Results

**Fig. 1:**
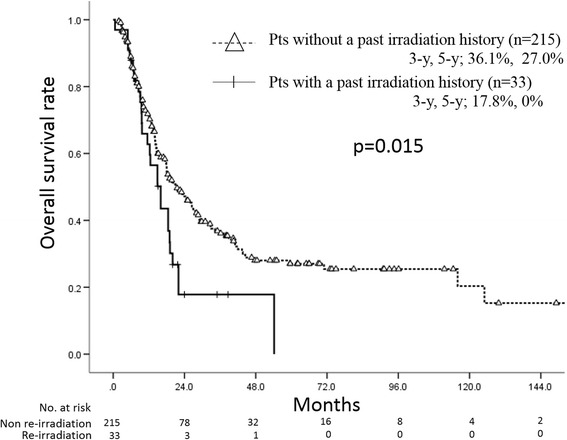
There was a significant difference between overall survival rates in patients with a past irradiation history and in patients without a past irradiation history (Kaplan-Meier method)

**Fig. 2:**
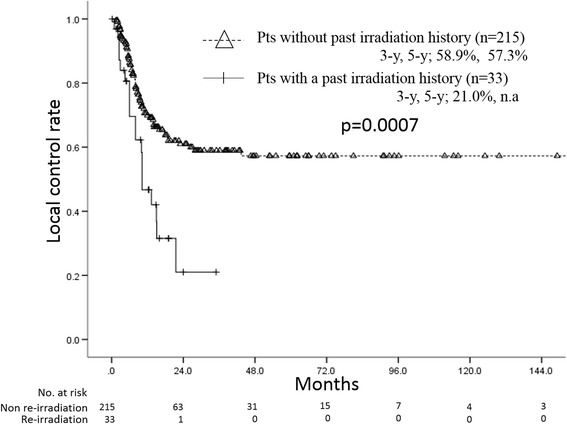
There was a significant difference between local control rate in patients with a past irradiation history and patients without a past irradiation history (Kaplan-Meier method)

Patients’ characteristics are shown in Table 1. Median maximum lymph node diameter was 22 mm (range, 5–106 mm). Histological diagnosis in all of the patients in whom re-irradiation was performed was squamous cell carcinoma. Median total radiation dose for oligo-recurrence was 60 Gy (range, 18–70 Gy). Of the 33 patients, 7 patients received definitive radiotherapy with median dose of 60 Gy (range, 50–60 Gy) as an initial treatment for esophageal cancer, 9 patients received adjuvant radiotherapy with median dose of 40 Gy (range, 40–60 Gy), and the others received neoadjuvant radiotherapy with median dose of 30 Gy (range, 30–40 Gy). Eleven of the 33 patients with a past irradiation history underwent re-irradiation by a hyperfractionation method with 1.2 Gy/fraction. Therefore, the median calculated biological effective dose using the Linear-Quadratic (LQ) model with α/β = 10 Gy (BED10) in patients in whom re-irradiation was performed was significantly lower than the median BED10 in others (Mann-Whitney U test, *p* < 0.001). All of the patients with a past irradiation history received involved field radiation therapy. There was no difference factor except for BED10, histology and irradiation field between patients with a past irradiation history and patients without a past irradiation history. Twenty-nine of the 33 patients received concurrent chemotherapy with re-irradiation. The regimen included an FP regimen (5-fluorouracil and cisplatin) in 15 patients, nedaplatin plus 5-fluorouracil in 12 patients, S1 alone in 1 patient, docetaxel alone in 1 patient. The median observation periods in all patients and seven surviving patients in whom re-irradiation was performed were 14.9 months and 21.7 months, respectively. The 3-year and 5-year overall survival rates in the 33 patients with a past irradiation history were 17.9% (95% confidence interval (C.I.) = 3.4–32.4%) and 0%, respectively, with a median survival period of 16.0 months (95% C.I. = 7.0–17.6) (Fig. 1). The 3-year and 5-year overall survival rates in 215 patients without a past irradiation history were 36.7% (95% C.I. = 29.4%–44.0%) and 26.9% (95% C.I. = 19.8%–34.0%), respectively, with a median survival period of 21.5 months (95% C.I. = 16.4–26.6). There was a significant difference between survival rates in the two groups (log-rank test, *p* = 0.015). The 3-year local control rates in the 33 patients with a past irradiation history and that in the 215 patients without a past irradiation history were 21.0% (95% C.I. = 0%–42.0%) and 58.9% (95% C.I. = 50.9%–66.9%), respectively (Fig. 2). There was a significant difference between local control rates in the two groups (log-rank test, *p* = 0.0007).

**Table 1:** Patients’ characteristics

		With RT history (*n* = 33)	Without RT history (*n* = 215)	
Age				n.s.
	Median	66 y-o	66 y-o	
Pathological stage (UICC 7th)				n.s.
	I–II	13	111	
	III–IV	19	101	
	Unknown	1	4	
Histology				*p* < 0.001 (Chi-squared test)
	SCC	33	209	
	Others	0	6	
Performance status (ECOG)				n.s.
	0–1	26	199	
	2–3	4	16	
	Unknown	3	0	
Tumor diameter				n.s.
	Median	27 mm	22 mm	
Disease-free interval				n.s.
	Median	7.3 months	11.7 months	
BED10				*p* < 0.001 (Mann-Whitney U test)
	Median	67.2 GyBED	72.0 GyBED	
Chemotherapy				n.s.
	+	29	180	
	−	4	35	
Irradiation field				*p* < 0.001 (Chi-squared test)
	Involved	33	154	
	Elective nodal	0	61	

Abbreviations: *RT* radiotherapy, *ECOG* Eastern Cooperative Oncology Group, *UICC* Union for International Cancer Control, *BED10* biological effective dose with α/β = 10 Gy, *n.s* not significant

One patient in whom re-irradiation was performed died from treatment-related gastric hemorrhage. There was no grade 3 or higher non-hematologic toxicity in patients with a past irradiation history other than that patients. In patients with overlapping irradiation fields, the median cumulative BED (range) for spinal cord, duodenum and membranous portion of the trachea using the LQ model with α/β = 3 Gy (BED3) was 70.2 GyBED (63–84.3), 134 GyBED (67.7–193.3) and 134 GyBED (95.4–134), respectively. On the other hand, grade 5 toxicity including pleural effusion, mediastinal-bronchial fistula, drug-induced interstitial pneumonia, and esophageal bleeding occurred in 4 of the 215 patients without a past irradiation history. Grade 4 toxicity including cardiac tamponade, hyperglycemia, esophagobronchial fistula, fistula of a gastric tube (2 cases) occurred in 5 of the 215 patients without a past irradiation history, and grade 3 toxicity including anastomotic stenosis and pleural effusion occurred in 2 of the 215 patients.

## Discussion

To the best of our knowledge, this is the first report showing that overall survival rate and local control rate in patients with oligo-recurrence in lymph nodes from esophageal cancer who had a past irradiation history were worse than those in patients without a past irradiation history. Even compared with past reports [5–9], the results for patients with a past irradiation history are poor (Table 2). There have been few reports on re-irradiation for recurrent esophageal cancer. However, results of re-irradiation in the head and neck region have been reported. It is thought that recurrent head and neck cancer may be more radioresistant than the primary tumors [10]. In intrathoracic recurrent non-small cell lung cancer, McAvoy et al. showed that a higher EQD2 as a re-irradiation dose was associated with improved overall survival [11]. One of the reasons why local control rate in patients with a past irradiation history was worse might be that patients with a past irradiation history were treated by less BED10 than patients without a past irradiation history due to concerns about potential toxicity of re-irradiation. We should probably use a higher BED in patients with a past irradiation history because it is assumed that recurrent esophageal cancer in patients with a past irradiation history is more radioresistant than that in patients without a past irradiation history.

**Table 2:** Literature review of treatment results for oligo-recurrence from esophageal cancer

Author	Year	No.	Method	3-year OS
Nakamura [6]	2008	22	CRT	24%
Maruyama [7]	2011	23	RT or CRT	31%
Jingu [8]	2012	30	CRT	38.4%
Bao [9]	2013	83	CRT	51.8%
Current study	2017	33	RT or CRT	17.8%

Abbreviations: *CRT* chemoradiotherapy, *RT* radiotherapy

Re-irradiation for recurrence after definitive radiotherapy or additional radiotherapy must be risky; however, the results of the present study indicated that toxicity of re-irradiation for oligo-recurrent esophageal cancer was acceptable. It is true that gastric hemorrhage in the re-irradiation group occurred at 18 Gy with a conventional fraction, but there is the possibility that it was caused by tumor invasion. Furthermore, considering that grade 3 or higher toxicity occurred in 11 of the 215 patients without irradiation history, at least severe toxicity was not more frequent than that in patients without a past irradiation history. Further investigation is needed to determine the appropriate irradiation dose and schedule. Qi et al. reported that the volume percent of the gastric tube receiving at least 50 Gy (V_50_) was strongly associated with the degree of toxicity [12]. The reason for the small number of cases of severe toxicity in the present study might be that involved field radiation therapy was performed in all patients who received re-irradiation. By using an involved field, it might be possible to use a higher BED safely even in patients with a past irradiation history. Jingu et al. showed by matched-pair analysis that elective nodal irradiation was not necessary in chemoradiotherapy for postoperative loco-regional recurrent esophageal cancer [13]. It is thought that the poor prognosis in patients who underwent re-irradiation was not due to the difference in irradiation field.

In the present study, almost all of the patients received 3D–conformal radiotherapy. In the near future, it might be possible to deliver a sufficient radiation dose without increasing toxicity due to IMRT or proton therapy. However, it should be noted that cases of esophageal or anastomotic recurrence were not included in this study. There are some reports of severe toxicity (e.g., ulcer, perforation) of re-irradiation for the esophagus even with IMRT or proton therapy. Kim et al. reported that grade 5 tracheoesophageal fistula occurred in 3 of 10 patients with recurrent esophageal cancer treated with re-irradiation [14].

The limitations of this study were its small sample size and retrospective analysis. It is necessary to perform prospective study with a much larger number of patients to determine the effectiveness and toxicity of re-irradiation for oligo-recurrence in lymph nodes from esophageal cancer.

## Conclusions

Results in the present study suggested that re-irradiation for oligo-recurrence in lymph nodes from esophageal cancer treated by definitive radiotherapy or by surgery with additional radiotherapy radiotherapy might be acceptable but unsatisfactory.

## Abbreviations

BED10: Biological effective dose using the Linear-Quadratic (LQ) model with α/β = 10 Gy
BED3: Biological effective dose using the Linear-Quadratic (LQ) model with α/β = 3 Gy
C.I.: Confidence interval
CTCAE: Common Terminology Criteria for Adverse Events
DFI: Disease-free interval
EQD2: Equivalent dose in 2 Gy fractions
IMRT: Intensity-modulated radiotherapy
